# Pathologic Findings in MRI-Guided Needle Core Biopsies of the Breast in Patients with Newly Diagnosed Breast Cancer

**DOI:** 10.4061/2011/613285

**Published:** 2010-12-01

**Authors:** K. P. Siziopikou, P. Jokich, M. Cobleigh

**Affiliations:** ^1^Department of Pathology, Feinberg School of Medicine, Northwestern University, Chicago, IL 60611-3008, USA; ^2^Department of Radiology, Rush University Medical Center, Chicago, IL 60612, USA; ^3^Department of Medical Oncology, Rush University Medical Center, Chicago, IL 60612, USA

## Abstract

The role of MRI in the management of breast carcinoma is rapidly evolving from its initial use for specific indications only to a more widespread use on all women with newly diagnosed early stage breast cancer. However, there are many concerns that such widespread use is premature since detailed correlation of MRI findings with the underlying histopathology of the breast lesions is still evolving and clear evidence for improvements in management and overall prognosis of breast cancer patients evaluated by breast MRI after their initial cancer diagnosis is lacking. In this paper, we would like to bring attention to a benign lesion that is frequently present on MRI-guided breast biopsies performed on suspicious MRI findings in the affected breast of patients with a new diagnosis of breast carcinoma.

## 1. Introduction

For patients with newly diagnosed breast carcinoma, evaluation of the extent of the disease in the breast is of paramount importance in planning appropriate surgical therapy. Magnetic resonance imaging (MRI) plays an ever increasing role in the evaluation of additional areas in the affected breast deemed suspicious but indeterminate by other radiologic modalities. Technical developments such as MRI with high spatial resolution, special breast coils, dynamic kinetic imaging techniques, and intravenous contrast agents contribute to enhanced diagnosis of breast abnormalities. While excitement about the role of this modality in improving the planning of surgical treatment of breast cancer patients is increasing [1–6], many areas of uncertainty remain, especially related to the clinical importance of additional lesions that are detected by the use of MRI [7–9]. In this study we evaluated the pathologic findings in MRI-guided needle core biopsies of the breast obtained from other suspicious areas in the affected breast of patients with a new diagnosis of breast carcinoma.

## 2. Materials and Methods

Our study population consisted of 44 MRI-guided needle core breast biopsies performed on 40 patients with newly diagnosed breast carcinoma at Rush University Medical Center, Chicago, IL, USA between May 2007 and July 2008. Histologic findings of these biopsies were reviewed and recorded. Patient age ranged from 36 to 77 years (average: 52 years).

## 3. Results

**Figure 1:**
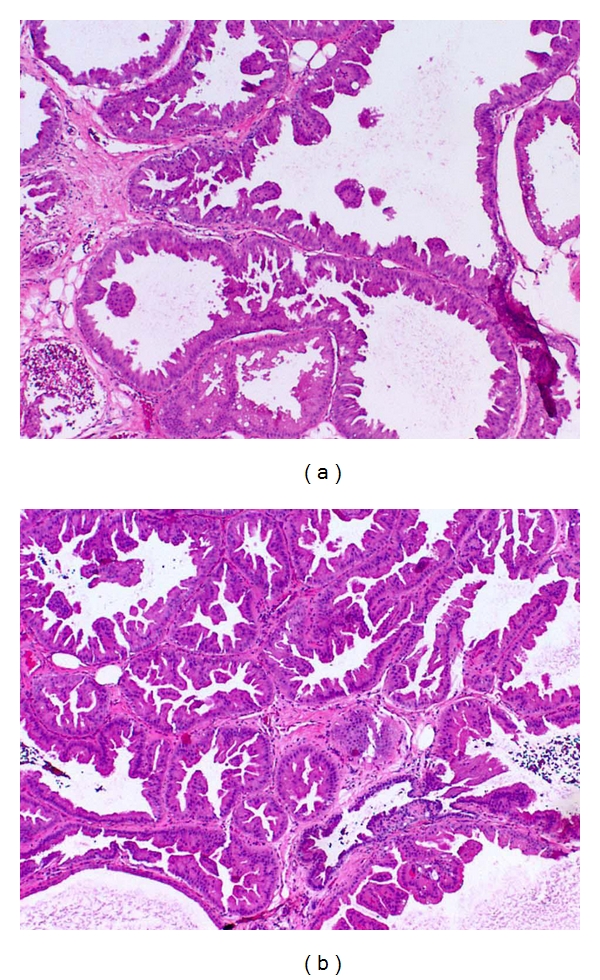
Representative examples of MRI-guided breast needle core biopsies showing a characteristic benign complex multicystic lesion lined by apocrine metaplastic epithelium, called “cystic apocrine metaplasia.” This benign lesion was seen in 38% (11/29) of the benign MRI-guided needle core biopsies in this series.

Overall, 9/44 (20.4%) of our MRI-guided breast biopsies were malignant, 29/44 (66%) were benign, and 6/44 (13.6%) showed atypia (Table 1). Of the 9 malignant cases, 4 were infiltrating ductal carcinomas with tubular features, 2 infiltrating lobular carcinomas, and 3 ductal carcinoma in situ lesions (Table 2). Of the 6 atypical cases, 2 were atypical ductal hyperplasia (ADH), 2 were atypical lobular hyperplasia (ALH), and 2 showed areas of columnar cell hyperplasia with atypia (Table 3). Of interest, more than one third of our benign cases (11/29, 38%) consisted of a specific complex multicystic lesion lined by apocrine metaplastic epithelium, a lesion we called “cystic apocrine metaplasia” (Figure 1).

## 4. Discussion

During the last few years there has been a heightened interest in the application of magnetic resonance imaging (MRI) in the management of breast cancer. Currently, MRI is used as a supplemental tool to complement conventional methods of radiologic and ultrasonographic breast evaluation. A number of appropriate indications for the clinical use of MRI in breast cancer diagnosis and management include clarification of questionable findings on mammography, evaluation and accurate staging of breast tumors in dense breasts, accurate evaluation of specific subtypes of breast carcinomas such as infiltrating lobular carcinoma, assessment of response to preoperative chemotherapy, diagnosis of occult primary breast tumors presenting with axillary nodal involvement, and surveillance programs assessing high-risk patients such as breast cancer gene carriers or patients with a history of chest irradiation [1–6]. 

However, as the use of MRI at the time of new diagnosis of early stage breast cancer is quickly becoming a new standard of care, there is heightened concern that routine use of MRI for preoperative staging may lead to more extensive surgery, while solid evidence for improvement of surgical management, improved rates of local control, or improved overall prognosis is lacking [8–12]. In addition, while in a number of studies the detection of additional foci of breast cancer by MRI in the affected breast is reported to be around 16% (ranging from 6 to 34%) [12, 13], only limited information exists on the histology of the additional lesions that MRI testing falsely identifies as worrisome [14].

In this paper, we report that MRI-guided needle core biopsies of separate lesions in the affected breast of patients with newly diagnosed breast carcinoma show additional foci of malignancy in 20% of cases. A high percentage (66%) of these additional suspicious areas by MRI are benign by histologic examination. More specifically, MRI-guided needle core biopsies seem to target a characteristic complex multicystic lesion lined by apocrine metaplastic epithelium, in over one third of the cases, a lesion we called “cystic apocrine metaplasia.” Our findings suggest that MRI-guided core biopsies result in an important change of detection of additional foci of malignancy in a significant number of cases. In addition, MRI-directed needle core biopsies often target benign lesions with specific histopathologic characteristics, namely, a characteristic complex multicystic lesion lined by apocrine epithelium, the so-called “cystic apocrine metaplasia.” We suggest that awareness of the correlation of this benign lesion with abnormal MRI findings by radiologists will be helpful as they evaluate breast MRI studies of patients with newly diagnosed early stage breast carcinoma.

Ongoing carefully controlled studies comparing the strengths and weaknesses of breast MRI to those of conventional breast imaging in different clinical scenarios, as well as detailed correlation of MRI findings to underlying histopathology of breast lesions, are urgently needed in order to clearly define diagnostic criteria for widespread MRI use.

## Figures and Tables

**Table 1:** Histologic findings of MRI-guided needle core biopsies.

Histology	No. of patients (*n* = 44)
Malignant	9 (20.4%)
Atypical	6 (13.6%)
Benign	29 (66%)

**Table 2:** Histologic findings of MRI-guided needle core biopsies that showed malignancy.

Malignant histology	No. of patients (*n* = 9)
IDC	4
ILC	2
DCIS	3

**Table 3:** Histologic findings of MRI-guided needle core biopsies that showed atypia.

Atypical histology	No. of patients (*n* = 6)
ADH	2
ALH	2
CCH with atypia	2

## References

[1] Liberman L (2004). Breast cancer screening with MRI—what are the data for patients at high risk?. *New England Journal of Medicine*.

[2] Kriege M, Brekelmans CTM, Boetes C (2004). Efficacy of MRI and mammography for breast-cancer screening in women with a familial or genetic predisposition. *New England Journal of Medicine*.

[3] Gundry KR (2005). The application of breast MRI in staging and screening for breast cancer. *Oncology*.

[4] Smith RA (2007). The evolving role of MRI in the detection and evaluation of breast cancer. *New England Journal of Medicine*.

[5] Mann RM, Hoogeveen YL, Blickman JG, Boetes C (2008). MRI compared to conventional diagnostic work-up in the detection and evaluation of invasive lobular carcinoma of the breast: a review of existing literature. *Breast Cancer Research and Treatment*.

[6] Teller P, Jefford VJ, Gabram SGA, Newell M, Carlson GW (2010). The utility of breast mri in the management of breast cancer. *Breast Journal*.

[7] Baltzer PAT, Benndorf M, Dietzel M, Gajda M, Runnebaum IB, Kaiser WA (2010). False-positive findings at contrast-enhanced breast MRI: a BI-RADS descriptor study. *American Journal of Roentgenology*.

[8] McCaffery KJ, Jansen J (2010). Pre-operative MRI for women with newly diagnosed breast cancer: perspectives on clinician and patient decision-making when evidence is uncertain. *Breast*.

[9] Solin LJ (2010). Counterview: pre-operative breast MRI (magnetic resonance imaging) is not recommended for all patients with newly diagnosed breast cancer. *Breast*.

[10] Houssami N, Hayes DF (2009). Review of preoperative magnetic resonance imaging (MRI) in breast cancer: should MRI be performed on all women with newly diagnosed, early stage breast cancer?. *CA Cancer Journal for Clinicians*.

[11] Katipamula R, Degnim AC, Hoskin T (2009). Trends in mastectomy rates at the Mayo Clinic Rochester: effect of surgical year and preoperative magnetic resonance imaging. *Journal of Clinical Oncology*.

[12] Morrow M, Harris JR (2009). More mastectomies: is this what patients really want?. *Journal of Clinical Oncology*.

[13] Houssami N, Ciatto S, Macaskill P (2008). Accuracy and surgical impact of magnetic resonance imaging in breast cancer staging: systematic review and meta-analysis in detection of multifocal and multicentric cancer. *Journal of Clinical Oncology*.

[14] Tozaki M (2004). Interpretation of breast MRI: correlation of kinetic and morphological parameters with pathological findings. *Magnetic Resonance in Medical Sciences*.

